# Differential Expression of lncRNAs and miRNAs Between Self-Rooting Juvenile and Donor Clones Unveils Novel Insight Into the Molecular Regulation of Rubber Biosynthesis in *Hevea brasiliensis*

**DOI:** 10.3389/fpls.2021.740597

**Published:** 2022-01-05

**Authors:** Hui-Liang Li, Ying Wang, Dong Guo, Jia-Hong Zhu, Shi-Qing Peng

**Affiliations:** Key Laboratory of Biology and Genetic Resources of Tropical Crops, Ministry of Agriculture, Institute of Tropical Bioscience and Biotechnology, Chinese Academy of Tropical Agricultural Sciences, Haikou, China

**Keywords:** *Hevea brasiliensis*, self-rooted juvenile clones, long noncoding RNA, miRNA, rubber biosynthesis

## Abstract

The rubber tree (*Hevea brasiliensis* Muell. Arg.) is a tropical tree species that produce natural rubber. Self-rooted juvenile clones (SRJCs) are novel rubber tree planting materials developed through primary somatic embryogenesis. SRJCs have a higher rubber yield compared with donor clones (DCs). The molecular basis underlying increased rubber yield in SRJCs remains largely unknown. Here, the latex from SRJCs and DCs were collected for strand-specific and small RNA-seq methods. A total of 196 differentially expressed long noncoding RNAs (DELs), and 11 differentially expressed microRNAs were identified in latex between SRJCs and DCs. Targeted genes of DELs were markedly enriched for various biological pathways related to plant hormone signal transduction, photosynthesis, glutathione metabolism, and amino acids biosynthesis. DELs probably acted as cis-acting regulation was calculated, and these DELs relevant to potentially regulate rubber biosynthesis, reactive oxygen species metabolism, and epigenetic modification. Furthermore, the DELs acting as microRNA targets were studied. The interaction of microRNA and DELs might involve in the regulation of natural rubber biosynthesis.

## Introduction

Natural rubber, an important industrial raw material, is widely used to produce tires and medical gloves (Cornish, 2017). Natural rubber production is derived entirely from the rubber tree (*Hevea brasiliensis* Muell. Arg.; Cherian et al., 2019). The rubber tree is a type of cross-pollinating plant. High-yielding varieties of rubber trees are bred through cross programs. The rubber tree is propagated by grafting axillary buds onto rootstocks grown from cross-pollinated seeds to preserve the desirable characters of an elite variety (Chandrashekar et al., 1997). Given the interactions between rootstock and scion, budding clones often show remarkable intraclonal variations in growth potential and rubber yield (Clément-Demange et al., 2007).

Novel rubber tree planting materials, called self-rooted juvenile clones (SRJCs), are developed through the somatic embryogenesis pathway (Wang et al., 1980; Chen et al., 1998, 2002). SRJCs have their own roots and juvenile characters. SRJCs have the fine characteristics of fast growth, higher laticifer number, high rubber yield, and strong stress resistance compared with donor clones (DCs; Chen et al., 1998, 2002; Yuan et al., 1998) and are likely to become a new generation of planting materials in the future (Hua et al., 2010). However, the mechanism underlying the difference between SRJCs and DCs, especially the mechanism of increased rubber yield in SRJCs remains largely unknown. Initially, a limited number of reports revealed that SRJCs have a high laticifer number and good rubber production and expulsion properties (Chen et al., 1998, 2002; Yuan et al., 1998; Hua et al., 2010). Recently, understanding the molecular mechanism of increased rubber yield in SRJCs has been given attention. Some studies have shown differentially expressed genes (DEGs) and proteins in the latex between SRJCs and DCs. These genes and proteins might participate in natural rubber biosynthesis and reactive oxygen metabolism, which may provide remarkable functions in the high-yielding rubber in SRJCs (Li et al., 2011a,b, 2014b, 2016a). Notably, some findings showed that the differential gene expression between SRJCs and DCs related to the modification of DNA methylation, histone methylation, and acetylation (Li et al., 2016a, 2017, 2020a).

Long noncoding RNAs (lncRNAs) belong to noncoding RNAs that are longer than 200 nucleotides (nt) in length. LncRNAs can regulate gene expression through sequence complementarity with RNAs or DNAs (Chekanova, 2015; Wang and Chekanova, 2017; Zhang et al., 2019a) and take part in chromatin remodeling, genomic imprinting, transcriptional interference, and transcriptional activation (Chekanova, 2015). Micro-RNA (miRNA) refers to small RNAs with lengths of 20–24 nt and regulates mRNA at transcriptional and post-transcriptional levels (Siddiqui et al., 2019). In plants, miRNAs have a significant regulatory role in a range of biological functions such as plant gene expression, abiotic and biotic stress, plant developmental events, etc. (Voinnet, 2009; Sunkar et al., 2012; Yu et al., 2017). The roles of lncRNAs and miRNAs in the rubber tree are yet to be fully investigated.

In a rubber tree, natural rubber is synthesized on the surface of rubber particles suspended in the latex (the cytoplasm of laticifers). The laticifers are specialized vessels located adjacent to the phloem of the rubber tree (Oh et al., 1999; Ko et al., 2003; Chow et al., 2007). Rubber molecules are accommodated in a specialized spherical organelle termed a rubber particle (RP). RPs comprise mainly a hydrophobic rubber molecule core (>90% of the total weight of RPs) surrounded by a lipid monolayer and membrane-bound proteins (Yamashita and Takahashi, 2020). After tapping, the severed laticiferous mantles expel a fraction of their contents in the form of latex. Latex contains about 35% of cis-1,4-polyisoprene, 60% of water, and 5% of a nonisoprene molecule, including proteins, lipids, carbohydrates, and minerals (Bottier, 2020). Although the non-isoprenes represent a minor part of the latex, some proteins such as cis-prenyltransferase, rubber elongation factor, and small rubber particle protein play a crucial role in the natural rubber biosynthesis (Oh et al., 1999; Ko et al., 2003; Chow et al., 2007). Because there is the absence of plasmodesmata or cytoplasmic connections between laticifers and their neighboring cells, the exuded latex should represent only the cytoplasmic contents of the laticifers uncontaminated by those of other cells. Since latex can be readily obtained in large quantities, it provides an opportunity to investigate the biochemical properties of a single, specialized cell type (Kush et al., 1990). Moreover, rubber biosynthesis takes place only in the latex, genes or proteins expressed in such tissues may involve rubber synthesis and regulation (Kush et al., 1990; Chow et al., 2007). In order to study the molecular mechanism associated with high yielding in SRJCs, we selected latex samples representing cytoplasmic content of laticiferous cells for RNA isolation to analyze the differential expressed lncRNAs (DELs) and microRNAs. Here, strand-specific RNA (ssRNA)-seq and small RNA (sRNA)-seq are performed on the latex of SRJCs and DCs. DELs and microRNAs in the latex between SRJCs and DCs are identified. The potential regulatory network of lncRNA–miRNA–mRNA is proposed. This study is helpful to understand the underlying mechanism of increased rubber yield in SRJCs and unveils new molecular regulation mechanisms of rubber biosynthesis in the rubber tree.

## Materials and Methods

### Plant Materials

Eight-year-old *H. brasiliensis* cultivar CATAS7-33-97 SRJCs and CATAS7-33-97 DCs were cultivated in the experimental field of Guangdong NongKen Tropical Crop Science Institute. Three SRJC groups (10 trees in each group) and three DC groups (10 trees in each group). Latex (10 ml from each plant) was collected from SRJCs or DCs. The latex was allowed to drop directly into liquid nitrogen in an ice kettle. After the latex drops in liquid nitrogen, small spherical particles are formed immediately. About 2–3 min, 10 ml of latex can be collected. Then the frozen latex was transferred to an RNAase-free tube and stored in liquid nitrogen. After all, samples were collected, they were returned to the laboratory and stored at −70°C or used immediately. Before RNA extraction, the samples in each group were mixed in equal quantities, and then RNA extraction buffer was added to start RNA extraction.

### Isolation of Latex RNA

The latex total RNA was isolated by the described method (Kush et al., 1990; Tang et al., 2007) with some modification. In brief, the latex powder was suspended in 10 ml RNA extraction buffer containing 1,000 mol.L^−1^ Tris–HCl (pH 8.0), 100 mmol.L^−1^ EDTA, 1.4 mol.L^−1^ LiCl, 20% (v/v) sodium dodecyl sulfate and 5% (v/v) mercaptoethanol, and the suspension was incubated at room temperate for 20 min, and was centrifuged at 12,000 × *g* for 10 min at 4°C. The lower liquid was transferred to a fresh tube. Then an equal volume of phenol/chloroform/isoamyl alcohol (25:24:1, v/v) was added and mixed evenly. The mixture was centrifuged at 12,000 × *g* for 10 min at 4°C. The supernatant was transferred to a fresh tube, an equal volume of phenol/chloroform/isoamyl alcohol (25:24:1, v/v) was added again and mixed evenly. The mixture was centrifuged at 112,000 × *g* for 15 min at 4°C. The supernatant was transferred to a fresh tube, and 1/3 vol of 8 mol.L^−1^ LiCl was added, and mixed well, and stored at 4°C overnight. The reaction mixture was centrifuged at 12,000 × *g* for 15 min at 4°C. The final pellet was washed with 70% (v/v) ethanol, air-dried, and dissolved in RNase-free water. Total RNA was monitored using agarose gel electrophoresis. The concentration and integrity of total latex RNA were measured using Qubit® RNA Assay Kit in Qubit® 2.0 Fluorometer (Life Technologies, CA, United States) and the RNA Nano 6,000 Assay Kit of the Bioanalyzer 2,100 system (Agilent Technologies, CA, United States).

### Library Construction and Sequencing

For lncRNA sequencing, the ribosomal RNA was depleted from the latex total RNA according to the Epicenter Ribo-Zero Gold Kit (Illumina, San Diego, United States). Then, six ssRNA-seq libraries (3 replicates in each sample) were constructed by the protocol of the Illumina TruSeq™ RNA sample prep Kit (Illumina, CA, United States) and were sequenced on the Illumina Hiseq 2,500 platform (LC-Bio, China). For sRNA sequencing, six sequencing libraries (3 replicates in each sample) were constructed following the protocol of the NEB Next Ultra-small RNA Sample Library Prep Kit for Illumina (NEB, Ipswich, MA, United States), and sequencing was carried out on the Illumina Hiseq 2,500 platform. Library construction and sequencing were carried out by Novogene Co., LTD (Beijing, China).

### LncRNAs and miRNA Identification

To generate clean data from ssRNA-seq, raw reads of FastQ format were firstly processed through in-house Perl scripts using the quality control software FastQC.^1^ In this step, clean data were obtained by removing reads containing adapter, reads containing more than 10% ploy-N, reads with average quality scores of each read of less than 20, and reads containing more than 40% of bases with quality scores less than 20. All obtained clean reads were mapped against the rubber tree genome (GenBank under the accession LVXX01000000, Tang et al., 2016) with STAR (v2.5.1b; Dobin et al., 2013) using the method of Maximal Mappable Prefix and assembled into transcripts by using the Cufflinks software (Trapnell et al., 2012). HTSeq v0.6.0 was used to count the reads numbers mapped to each gene (Anders et al., 2015). The raw count data were subsequently subjected to library-size normalization by edgeR software (Robinson et al., 2010). The expression level of transcripts was calculated using the Fragments Per Kilobase per Million mapped reads (FPKM).

According to a previously described method (Ding et al., 2019), lncRNAs were identified according to the following criteria: (i) transcripts that overlapped with known protein-coding genes on the same strand, transcripts with FPKM scores less than 0.5, and transcripts shorter than 200 nt were filtered; (ii) the transcripts with protein-coding potential were also discarded according to the evaluation of Coding-Potential Assessment Tool (Wang et al., 2013a), Coding Potential Calculator (Kong et al., 2007), and Coding-Non-Coding Index (Sun et al., 2013); and (iii) the transcripts with well-known protein domains were also removed based on the Pfam-hidden Markov models (Finn et al., 2008). The remaining transcripts were regarded as reliably expressed lncRNAs. DEGs, DELs between SRJCs and DCs were identified by DESeq2 by setting |log_2_fold-change| > 1 and *p* < 0.05 (Love et al., 2014).

For sRNA analysis, clean reads were obtained by removing contaminated reads, low-quality reads, and short sequences with base lengths less than 18 nt from raw data. Then, the clean reads were aligned to the GenBank (Release 209.0) and Rfam (Release 11.0) databases, and the reads annotated as small nucleolar RNA, small nuclear RNA, small cytoplasmic RNA, ribosomal RNA, and translocation RNA were removed. The remaining clean reads were mapped to the rubber tree reference genome (Tang et al., 2016) by using the Bowtie software (Langmead et al., 2009). The known miRNAs were identified by searching against miRbase (Release 22; Griffiths-Jones et al., 2006) and PNRD (Yi et al., 2015) databases. Novel miRNAs were identified according to their genome positions and hairpin structures predicted by Mireap v0.2 software (Wen et al., 2010; Friedlander et al., 2011). The microRNAs expression levels were estimated by TPM (transcript per million) through the following criteria (Zhou et al., 2010): Normalization formula: Normalized expression = mapped read count/Total reads ×10^6^. Differential expression miRNA (DEMs) between SRJCs and DCs was determined using the DESeq R package (1.8.3). The *p*-values were adjusted using the Benjamini and Hochberg method (Benjamini and Hochberg, 1995). Corrected p-value of 0.05 was set as the threshold for significantly differential expression by default.

### Target Gene Prediction of DELs

Identifying the targets of lncRNAs in *cis*- or *trans* regulation was implemented according to a previously described method (Wu et al., 2019; Fu et al., 2020). In brief, DELs and their nearby protein-encoding genes, which were located 100 kb downstream and upstream of DELs, were used to conduct co-expression analysis and identify the targets of lncRNAs in *cis*-regulation. DEL–DEG pairs that were nearly located and highly co-expressed were considered as *cis*-acting regulation. The target genes of trans-acting lncRNAs were identified by the correlation of expression levels. Pearson correlation coefficients (Pripp, 2018) between the expression of DELs and DEGs were calculated, and DEGs with an absolute value of the correlation coefficient of more than 0.95 were predicted as *trans*-target genes of DELs.

### Prediction of DELs as Precursors, Targets, and eTMs (Endogenous Target Mimics) of miRNAs

DELs as precursors of DEMs were identified by aligning DELs to miRbase (Release 22; Griffiths-Jones et al., 2006) by Blast. Those with identity higher than 90% were regarded as putative miRNA precursors. In addition, the software miRPara (Wu et al., 2011) was also used to predict DEM precursors from DELs.

To identify DELs that were targeted by DEMs, the DELs and DEMs data were predicted by psRNATarget (Dai et al., 2018) with expectation ≤3 based on perfect or near-perfect complementarity between the miRNAs and the target lncRNAs sequences. DELs acting as eTMs of DEMs were predicted by TAPIR (Bonnet et al., 2010) according to the following criteria (Wu et al., 2019): (i) perfect base pairing was required at the 2nd to 8th positions from the 5′ end of miRNA sequence; (ii) the total number of mismatches and G/U pairs within the miRNA and DEL pairing region (excluding the bulge) should be no more than three; (iii) a three-nucleotide bulge was only permitted at the 9th to 12th positions from the 5′ end of miRNA sequence.

### LncRNA–miRNA–mRNA Network Construction

Based on the above prediction results, the regulatory networks of DEL-DEG, DEM-DEG, and DEM-DEL were constructed visualized with Cytoscape (v3.8.0; Su et al., 2014). Subsequently, the interaction networks of DELs, DEMs, and DEGs were imported into Cytoscape for visualization (Su et al., 2014).

### Functional and Pathway Enrichment Analysis

The enrichment of the Gene Ontology (GO) terms^2^ and Kyoto Encyclopedia of Genes Genomes (KEGG) pathways^3^ in the target genes of DELs were performed by the cluster Profiler R package (Yu et al., 2012) setting *p* value <0.05 as significantly enriched.

### Quantitative PCR

cDNA was synthesized following the protocol of cDNA Synthesis Kit (TaKaRa, China). qRT-PCR was performed using SYBR Premix Ex Taq Kit (TaKaRa, China) on the Mx3005P Real-Time PCR System (Stratagene, United States). The *HbACT7* was used as the internal reference gene (Li et al., 2016a). The primers are listed in Supplementary Table S1. The expression of lncRNAs and mRNAs were normalized using a 2^−∆∆CT^ method (Schmittgen and Livak, 2008).

## Results

### Identification and Characterization of LncRNAs in Rubber Tree

**Figure 1:**
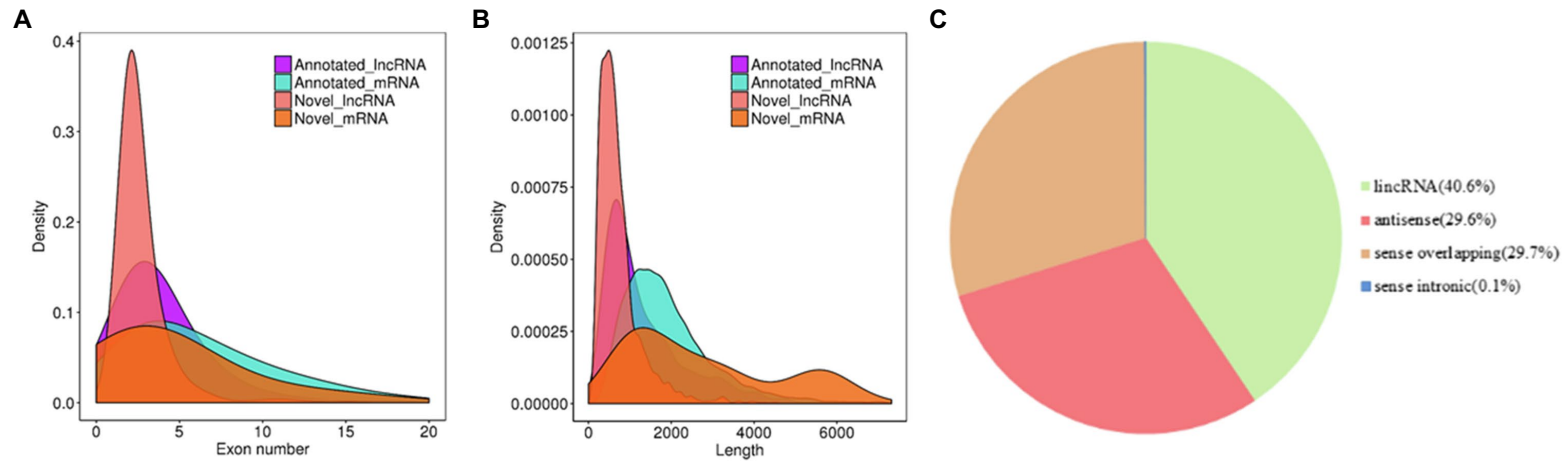
Features of rubber tree lncRNAs. **(A)** Exon number and **(B)** The density of transcript lengths in lncRNAs and mRNA. **(C)** The proportions of different types of lncRNAs.

The systematic identification of lncRNAs in rubber trees was performed in latex from SRJCs and DCs using ssRNA-seq. In total, 642 million clean reads were obtained by all ssRNA-seq data (Supplementary Table S2). A total of 48031 transcripts were obtained. Of these transcripts, 44,374 overlapping with 25,738 rubber tree protein-encoding genes were removed. A total of 3,657 lncRNAs were identified from the rubber tree, and 1,128 of these lncRNAs were novel lncRNAs (Supplementary Table S3). Approximately 50.4, 30.9, and 8.9% of lncRNAs had lengths of 200–600, 601–1,000, and 1,001–1,400 bp, respectively, and the remaining 10.8% of lncRNAs had a length of more than 1,400 bp (Figure 1A). Most (84.3%) lncRNAs contained two or three exons, and approximately 8.4, 2.8, and 1.7% of lncRNAs contained four, five, and six exons, respectively. The remaining 2.8% of lncRNAs contained more than six exons (Figure 1B). According to their genomic locations, 40.6% of lncRNAs were long intervening noncoding RNAs (lincRNAs), approximately 29.6 and 29.7% of lncRNAs were antisense and sense lncRNAs, and 0.1% of lncRNAs was overlapping lncRNAs, respectively (Figure 1C).

### DELs and Target Genes of DELs

**Figure 2:**
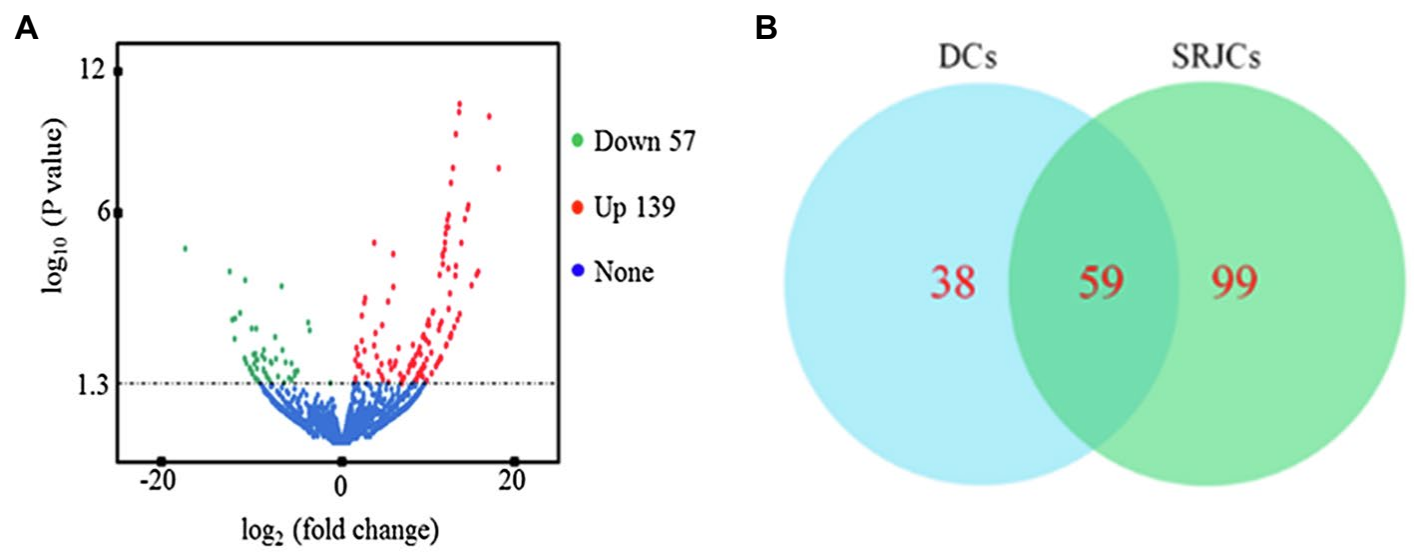
Differential expressed lncRNAs (DELs) between SRJCs and DCs. **(A)** Volcano plot showing DELs between SRJCs and DCs. **(B)** Venn diagram of DELs between SRJCs and DCs.

In total, 3,013 and 2,982 lncRNAs were identified in SRJCs and DCs, respectively. A total of 196 DELs, including 57 downregulated and 139 upregulated lncRNAs, were identified between SRJCs and DCs (Figure 2A). Among DELs, 38 lncRNAs were unique in DCS, and 99 lncRNAs were unique in SRJCs (Figure 2B).

**Figure 3:**
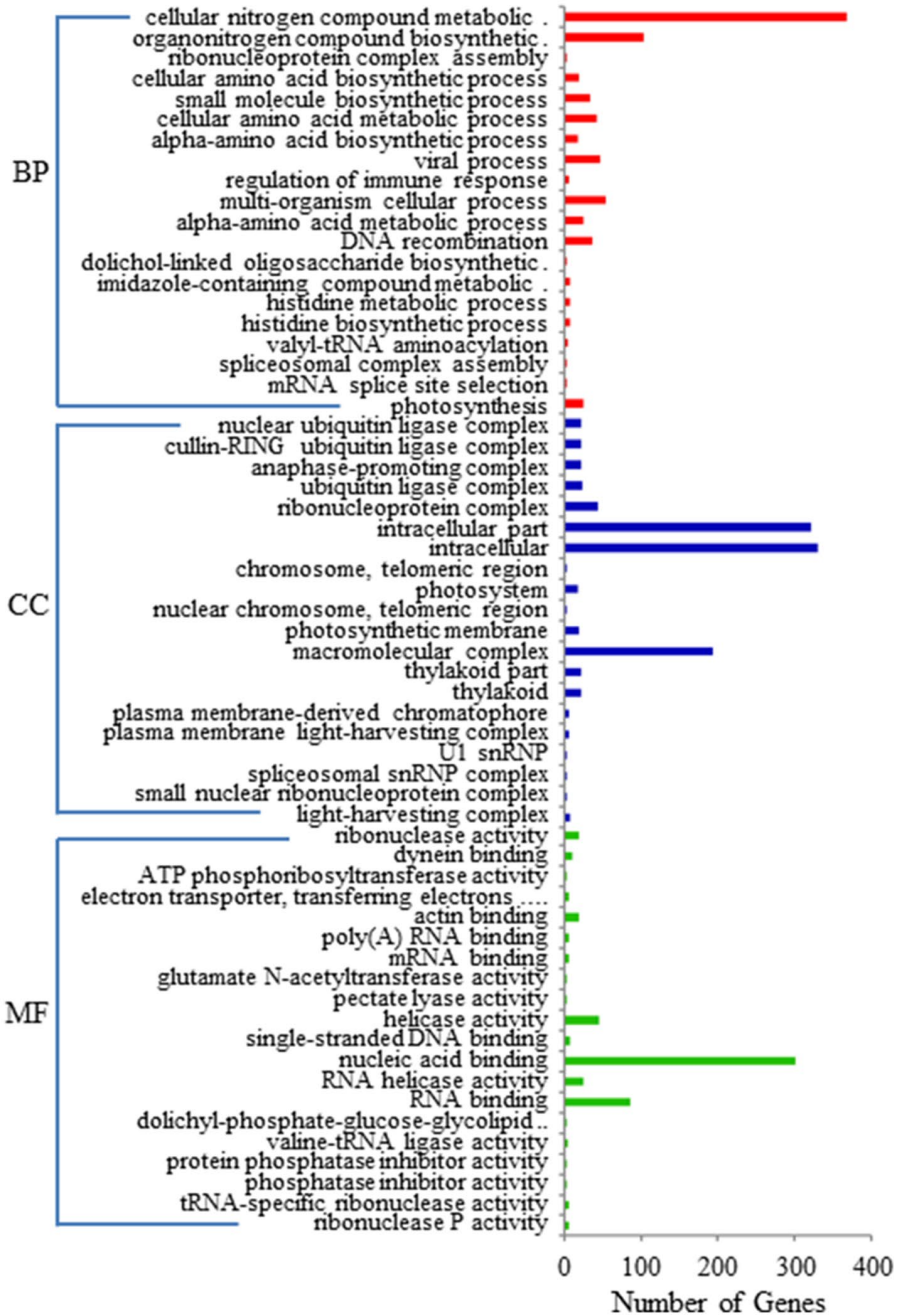
GO terms for the target genes of DELs between SRJCs and DCs.

**Figure 4:**
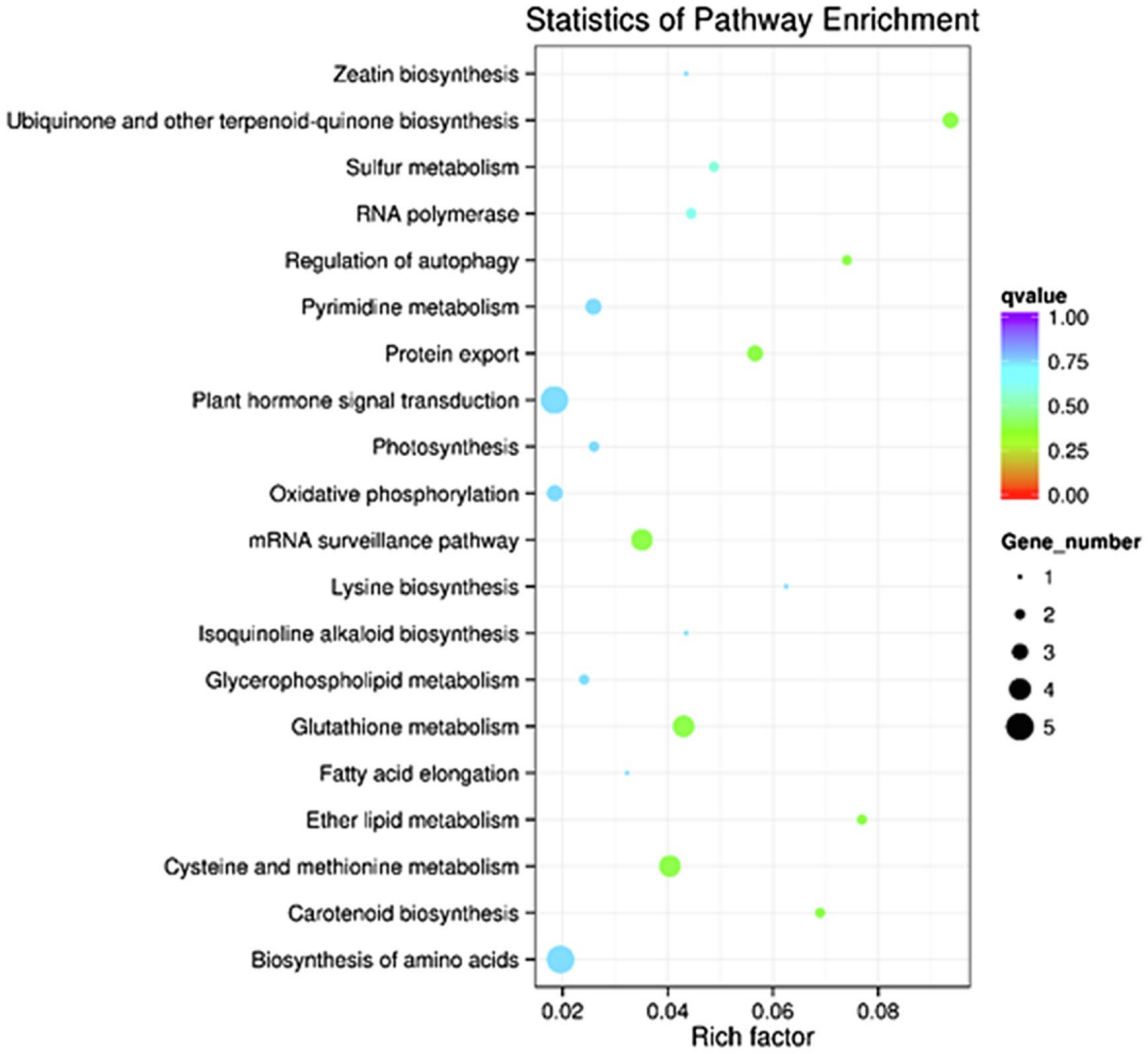
The enriched KEGG pathways for the target genes of DELs between SRJCs and DCs.

GO enrichment and KEGG pathway analyses were performed on the 1796 target genes of DELs. Under three GO categories, the term with the largest proportion in biological process was cellular nitrogen compound metabolic process (Figure 3). The analysis results of KEGG pathway enrichment showed that the target genes of DELs were associated with photosynthesis, plant hormone signal transduction, glutathione metabolism, oxidative phosphorylation, protein export, oxidative phosphorylation, and amino acids biosynthesis (Figure 4).

### DELs Act in Cis-Regulation

**Figure 5:**
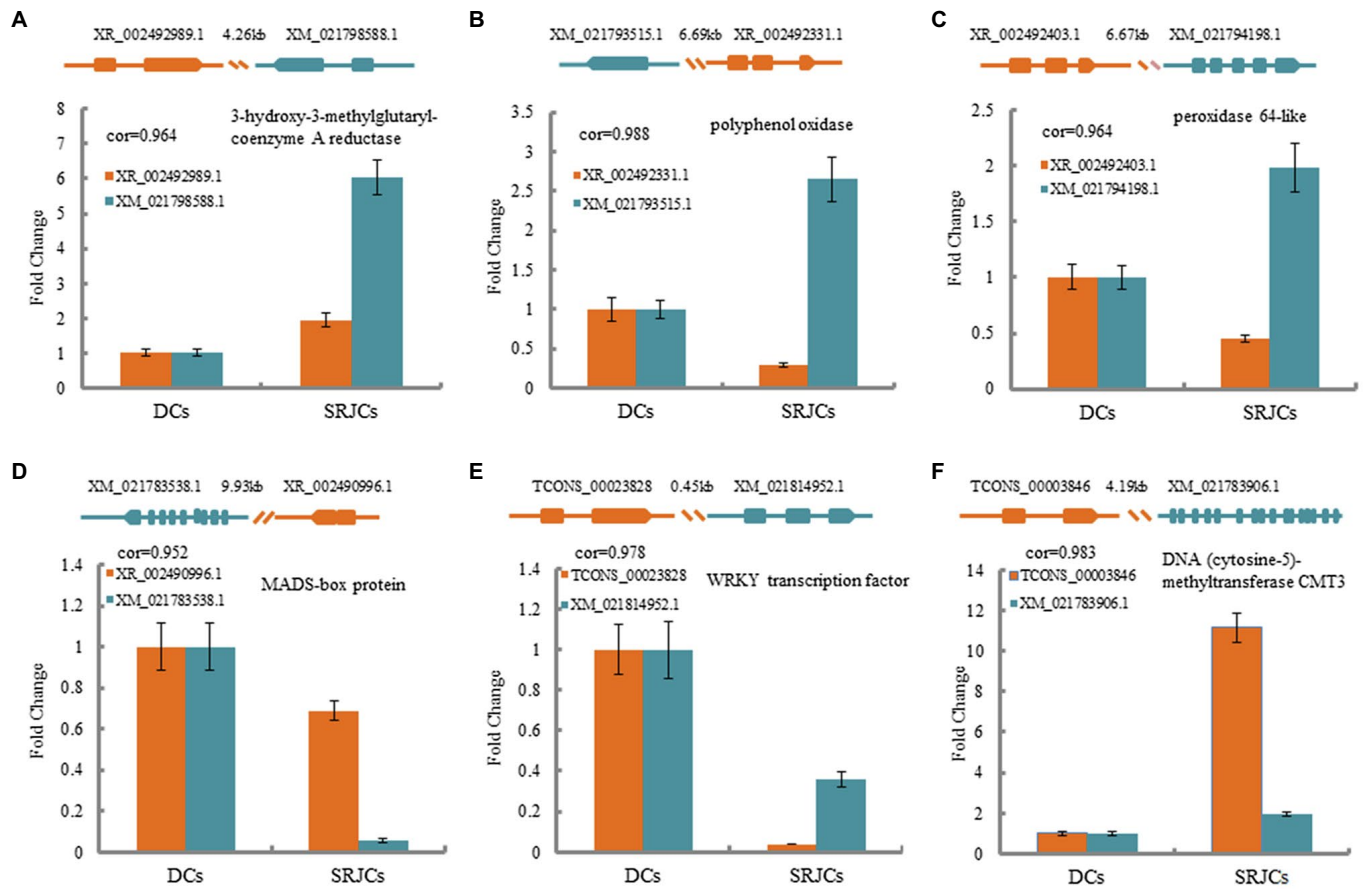
The expression fold change and structures of lncRNA–mRNA pairs in cis-acting regulation participating in natural rubber biosynthesis **(A)**, reactive oxygen species metabolism **(B,C)**, transcription factor **(D,E)**, and DNA methylation **(F)**.

A total of 122 lncRNA–mRNA pairs related to cis-acting regulation were obtained (Supplementary Table S4). Among these lncRNA–mRNA pairs, XR_002492989.1 was spaced 4.26 kb upstream of XM_021798588.1, which encoded a 3-hydroxy-3-methylglutaryl-coenzyme A reductase (HMGR) that catalyzes the conversion of HMG-CoA to mevalonic acid, which is a central step for the biosynthesis of a number of natural products (Figure 5A). XR_002492331.1 was located 6.69 kb upstream of XM_021793515.1, which encoded a polyphenol oxidase (Figure 5B), and XR_002492403.1 was located 6.67 kb upstream of XM_021794198.1, which encoded a peroxidase 64-like (Figure 5C), suggesting that XR_002492331.1 and XR_002492403.1 were related to reactive oxygen species metabolism. XR_002490996.1 was located 9.93 kb upstream of XM_021783538.1, which encoded a MADS-box transcription protein (Figure 5D), and TCONS_00023828 was located 0.45 kb upstream of XM_021814952.1, which encoded a WRKY transcription factor (Figure 5E). TCONS_00003846 was spaced 4.19 kb upstream of XM_021783906.1, which encoded a DNA (cytosine-5)-methyltransferase CMT3 that participated in the DNA methylation (Figure 5F). These results suggested that these cis-acting DELs might involve in the regulation of their adjacent genes connected with natural rubber biosynthesis, reactive oxygen species metabolism, transcription regulation, and DNA methylation.

### Expression Verification of Selected LncRNAs and Genes

**Figure 6:**
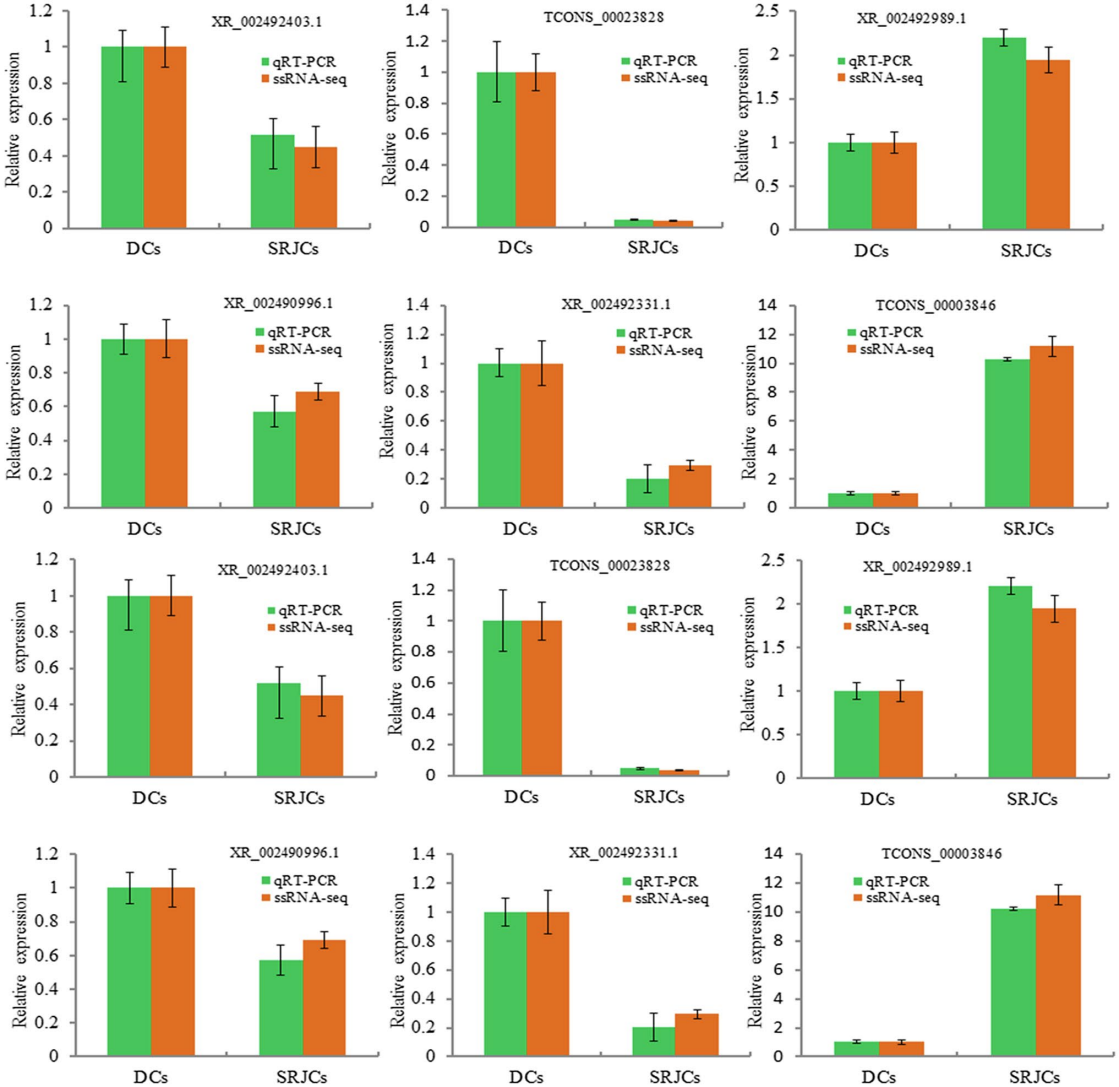
qPCR validation of mRNAs and lncRNAs.

A total of 8 DELs and 4 genes were randomly selected to examine the expression by qRT-PCR. qPCR results showed the expression of selected DELs and genes were highly consistent with ssRNA-seq data (Figure 6).

### sRNA Sequencing and Identification of miRNA

A total of 15,778,815 and 11,396,302 raw reads were obtained in SRJCs and DCs, respectively. After removing reads less than 18 nt, adapter sequences, and low-complexity sequences, averages of 3,354,892 and 3,867,160 unique reads were obtained (Supplementary Table S5). More than 80% of the sRNAs had lengths of 18–24 nt.

**Figure 7:**
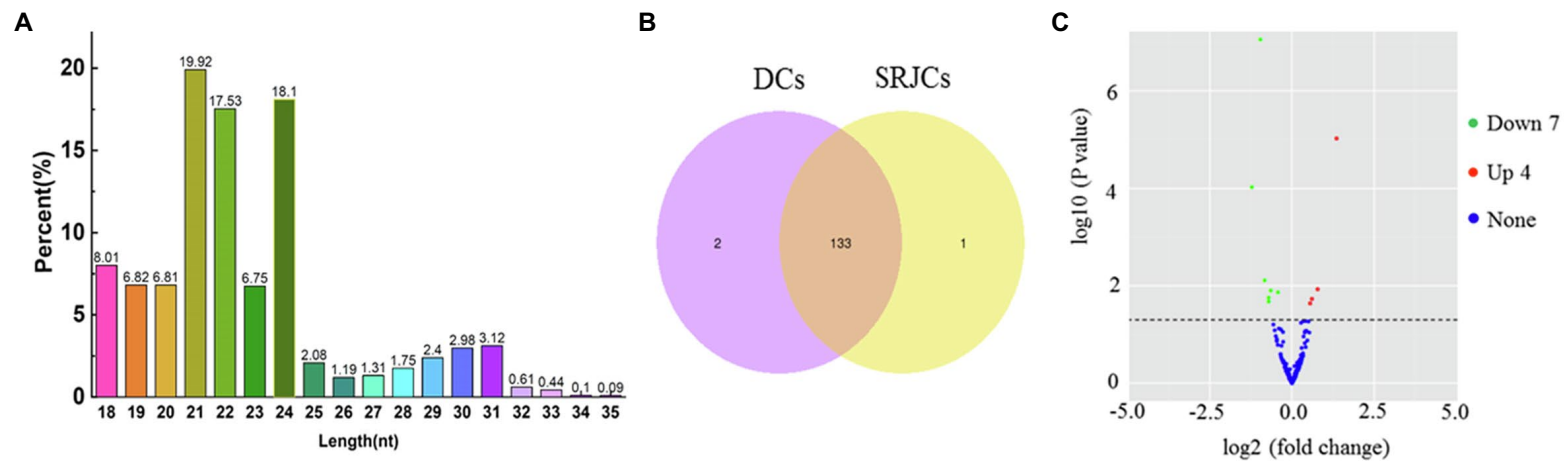
Features of miRNAs in SRJCs and DCs. **(A)** Frequency percentage of different miRNA in length. **(B)** Venn diagram of differentially expressed miRNAs between SRJCs and DCs. **(C)** Volcano plot showing differentially expressed miRNAs between SRJCs and DCs.

In total, 136 miRNAs, including 108 novel and 28 known miRNAs, were found in DCs and SRJCs. About 41.96, 29.23, and 14.61% of these miRNAs had lengths of 21, 24, and 22 nt, respectively (Figure 7A). Among 136 miRNAs, 133 were common to DCs and SRJCs, and two and one miRNAs were specific to DCs and SRJCs, respectively (Figure 7B). Eleven differential expression miRNAs, including four upregulated and seven downregulated miRNAs, were found between DCs and SRJCs (Figure 7C).

### LncRNA–miRNA–mRNA Network Construction

**Figure 8:**
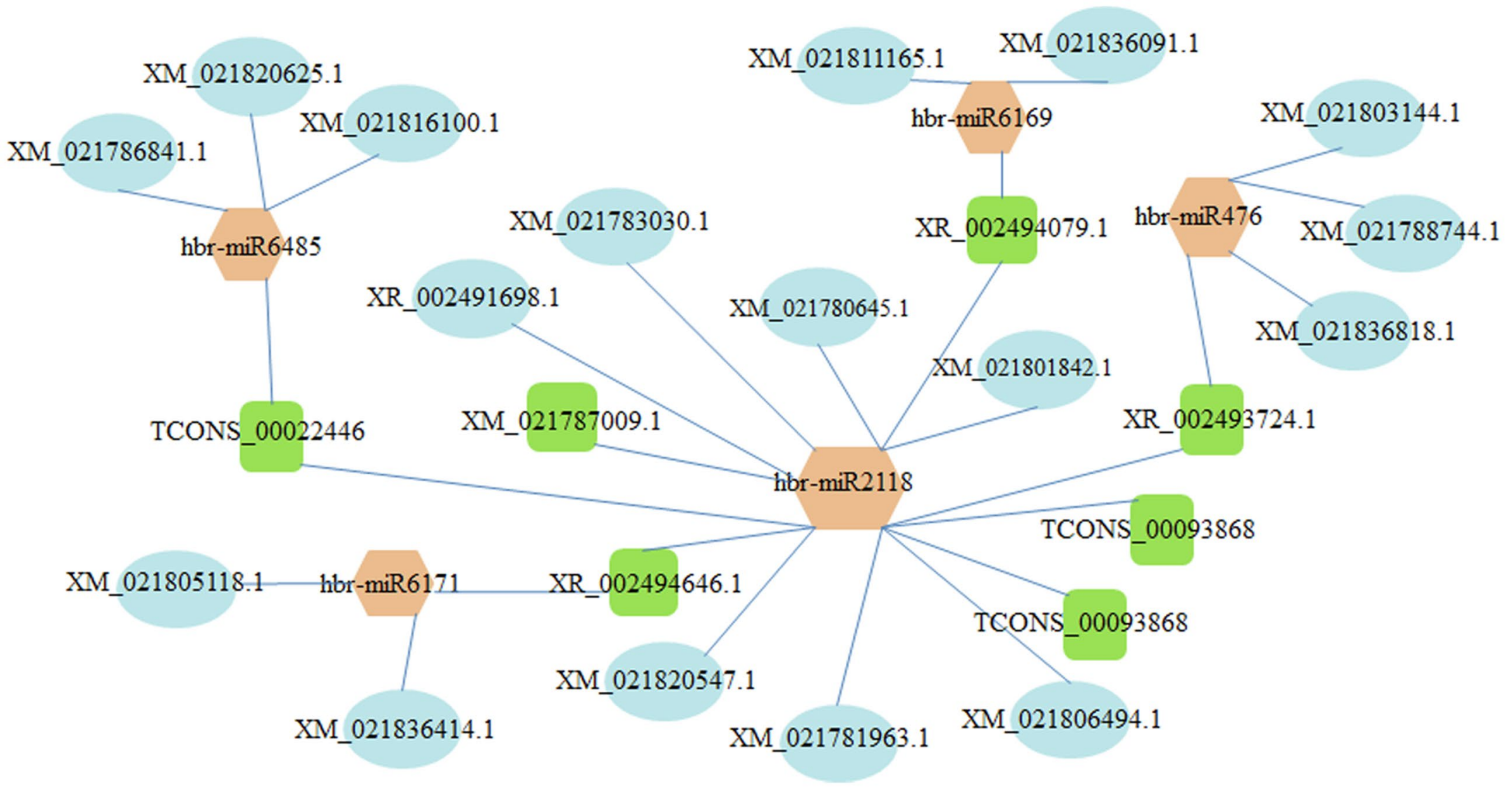
lncRNA–miRNA–mRNA network. Ovals, squares, and hexagons represent mRNAs, lncRNAs, and miRNAs.

The construction of the lncRNA-miRNA-mRNA network is helpful to understand the regulatory role of DELs in rubber trees. We constructed the lncRNA–miRNA–mRNA network based on the co-expression of mRNA and DELs, and DELs–miRNA interaction pairs. As shown in Figure 8, the lncRNA–miRNA–mRNA network contained 5 miRNAs (i.e., hbr-miR2118, hbr-miR47, hbr-miR6169, hbr-miR6171, and hbr-miR6485), 7 lncRNAs (e.g., TCONS_00093868, XR_002494646.1, and XR_002493724.1), and 23 mRNAs (e.g., XM_021806494.1 encoding small rubber particle protein (SRPP), XM_021836414.1 encoding superoxide dismutase, XM_021788744.1 encoding ethylene-responsive transcription factor ERF091, XM_021788744.1 and XM_021836818.1 encoding DNA (cytosine-5)-methyltransferase DRM2, and XM_021803144.1 encoding histone deacetylase 9 (Supplementary Table S7).

## Discussion

Although the natural rubber biosynthesis pathway has been identified (Sando et al., 2008; Tang et al., 2016), the molecular regulation of rubber biosynthesis is largely unknown (Chow et al., 2007; Yamashita et al., 2016; Deng et al., 2018). Progress has been made in the transcriptional regulation of natural rubber biosynthesis. Some transcription factors that regulate key genes of natural rubber biosynthesis have been proven. For example, HbWRKY1 and HbWRKY14 are identified to act as negative regulator of *HbSRPP*, encoding the SRPP participating in natural rubber biosynthesis (Wang et al., 2013b). The interaction of histone deacetylase (HbHDA3) with HbWRKY14 relieves the inhibition of *HbSRPP* expression by HbWRKY14-mediate (Li et al., 2020b). HbMADS4 negatively regulates the *HbSRPP* expression (Li et al., 2016b), whereas HbMYC2b upregulates the expression of *HbSRPP* (Guo et al., 2019). HbCZF1 positively regulates the expression of *hmg1*, which encodes HMGR (Guo et al., 2015). HblMYB19, HblMYB44, and HbWRKY27 positively regulate the expression of the farnesyl pyrophosphate synthase (FPS) gene *HbFPS1* (Wang et al., 2017; Qu et al., 2020). HMGR and FPS are key enzymes during natural rubber biosynthesis. HbRZFP1 downregulates the expression of *HRT2*, which encodes rubber transferase that is a key enzyme participating in natural rubber biosynthesis (Guo et al., 2018). Further study on the molecular mechanism of natural rubber biosynthesis is important for the genetic improvement of *H. brasiliensis*.

LncRNAs play a key regulatory role in gene expression in plants. To date, lncRNAs have not been reported in rubber trees. Here, 3,657 lncRNAs were systematically identified in a rubber tree by using ssRNA-seq. A total of 185 DELs were identified between DCs and SRJCs. The target genes of DELs were enriched for biological pathways linked to photosynthesis, plant hormone signal transduction, glutathione metabolism, and amino acids biosynthesis.

LncRNAs act as regulators to modulate gene expression. For example, in maize, lncRNA Vgt1 can depress the *ZmRap2* expression related to flowering time (Li et al., 2014a), and tomato lncRNA33732 activates *RBOH* expression and is involved in early defense responses (Cui et al., 2019). The latex productivity of a rubber tree depends mainly on the duration of latex flow after tapping and the capability of latex regeneration between two consecutive tappings (Chrestin et al., 1997). The duration of the flow is limited by the coagulation. The reactive oxygen species (ROS) production and ROS-scavenging in laticifers affect latex coagulation (Zhang et al., 2019b). In addition, transcription factors are actively implicated in the regulation of latex regeneration (Wang et al., 2013b; Li et al., 2016b, 2020a; Zhang et al., 2019b; Qu et al., 2020). In the present study, DELs acting in the cis-regulation were found. The genes regulated by lncRNAs were concerned with natural rubber biosynthesis and ROS metabolism. For example, XR_002492989.1 was located 4.26 kb upstream of XM_021798588.1 encoding HMGR. In addition, XR_002492331.1 was located 6.69 kb upstream of XM_021793515.1 encoding a polyphenol oxidase; and XR_002492403.1 was located 6.67 kb upstream of XM_021794198.1 encoding a peroxidase 64-like. HMGR is a rate-limiting enzyme participating in the biosynthesis of natural rubber (Chye et al., 1992; Guo et al., 2015). Polyphenol oxidase and peroxidase are the main sources of antioxidant metabolism in latex (Zhang et al., 2019b). The over-accumulation of ROS can lead to laticifer dysfunctions, thereby affecting latex flow and production in rubber trees (Chrestin et al., 1997). *HMGR*, *polyphenol oxidase*, and *peroxidase 64-like* were upregulated in self-rooting JCs (Figures 5A–C), suggesting that the self-rooting JCs provide a sufficient molecular basis for the increased rubber yielding, especially in the aspects of improved natural rubber biosynthesis, latex flow, and production. XR_002492989.1 XR_002492331.1, and XR_002492403.1 were upregulated in self-rooting JCs (Figures 5A–C), which was positively correlated with the expression of *HMGR*, *polyphenol oxidase*, and *peroxidase 64-like*, suggesting that these lncRNAs might play roles in increased rubber yield in SRJCs. Furthermore, XR_002490996.1 was spaced 9.93 kb downstream of XM_021783538.1, which encodes a MADS-box transcription factor, and TCONS_00023828 was located 0.45 kb upstream of XM_021814952.1, which encodes a WRKY protein. The MADS-box and WRKY transcription factor have been identified to negatively regulate the biosynthesis of natural rubber (Wang et al., 2013b; Li et al., 2016b, 2020b; Qu et al., 2020). XM_021783538.1 and XM_021814952.1 were down-regulated in self-rooting JCs (Figures 5D,E). The expression of XR_002490996.1 and TCONS_00023828 was negatively correlated with the expression of XM_021783538.1 and XM_021814952.1, suggesting that XM_021783538.1 and XM_021814952.1 might negatively regulate the biosynthesis of natural rubber in DCs and play a regulation role in increased rubber yield in SRJCs.

LncRNAs can function as miRNA targets except in cis- and trans-regulation (Wu et al., 2013; Fatica and Bozzoni, 2014; Paraskevopoulou and Hatzigeorgiou, 2016). XM_021806494.1, which encodes SRPP, is targeted by TCONS_00093868 and hbr-miR2118. SRPP is a key rubber particle protein in latex and implicates the natural rubber biosynthesis (Oh et al., 1999; Berthelot et al., 2014; Brown et al., 2017). The expression of hbr-miR2118 was upregulated in self-rooting JCs (Supplementary Table S7), TCONS_00093868 might participant in the regulation of natural rubber biosynthesis as hbr-miR2118 target that might play a regulation role in increased rubber yield in SRJCs. XM_021836818.1 encoding DRM2 and XM_021803144.1 encoding histone deacetylase 9 are targeted by XR_002493724.1 and hbr-miR476. Histone deacetylase and DRM2 play essential roles in epigenetic modifications (Pandey et al., 2002; Tian et al., 2005; Ashapkin et al., 2016; Zhang et al., 2018), and epigenetic modifications may lead to gene differential expression between self-rooting JCs and DCs (Li et al., 2016a, 2017, 2020a), indicating that XR_002493724.1 has essential role in differential gene expression between SRJCs and DCs as hbr-miR476 target. However, the functions of these lncRNAs need further verification and study.

## Conclusion

In this study, several novel lncRNAs were identified in rubber trees. Their basic features were studied, and their potential functions were predicted. The data were useful to study the roles of lncRNAs in regulating natural rubber biosynthesis. Specifically, lncRNAs TCONS_00093868 might be important candidates participating in the natural rubber production *via* the lncRNA–miRNA interaction.

## Data Availability Statement

The original contributions presented in the study are publicly available. This data can be found in the NCBI-SRA repository accession numbers SRR15205282 to SRR15205293. https://www.ncbi.nlm.nih.gov/sra/?term=SRR15205282; https://www.ncbi.nlm.nih.gov/sra/?term=SRR15205293.

## Author Contributions

H-LL: conceived, performed the experiments, and data analysis. YW: performed the experiments and data analysis. DG: performed the experiments. J-HZ: data analysis. S-QP: conceived the study and wrote the manuscript. All authors have read and approved the final manuscript.

## Funding

The project was supported by Hainan Provincial Natural Science Foundation of China (320RC712), National Natural Science Foundation of China (32171827), and National Key Research and Development Program of China (2018YFD1000502).

## Conflict of Interest

The authors declare that the research was conducted in the absence of any commercial or financial relationships that could be construed as a potential conflict of interest.

## Publisher’s Note

All claims expressed in this article are solely those of the authors and do not necessarily represent those of their affiliated organizations, or those of the publisher, the editors and the reviewers. Any product that may be evaluated in this article, or claim that may be made by its manufacturer, is not guaranteed or endorsed by the publisher.

## Footnotes

^1^ http://www.bioinformatics.babraham.ac.uk/projects/fastqc/

^2^ http://geneontology.org/

^3^ http://www.genome.jp/kegg/

## Supplementary Material

Supplementary Table S1 qRT-PCR primers used in this study.

Supplementary Table S2 Statistical data of the reads for six cDNA libraries.

Supplementary Table S3 Information of novel lncRNA.

Supplementary Table S4 Summary of 122 presented lncRNA–mRNA pairs in cis-acting regulation.

Supplementary Table S5 Statistical data of the reads of sRNA sequencing.

Supplementary Table S6 Summary of mRNAs or lncRNAs acting as miRNA targets.

Supplementary Table S7 Information of genes presented in the lncRNA–miRNA–mRNA network.
